# The Personal Sequence Database: a suite of tools to create and maintain web-accessible sequence databases

**DOI:** 10.1186/1471-2105-8-479

**Published:** 2007-12-18

**Authors:** Scott A Givan, Christopher M Sullivan, James C Carrington

**Affiliations:** 1 Center for Genome Research and Biocomputing, Oregon State University, Corvallis, Oregon, USA; 2 Department of Botany and Plant Pathology, Oregon State University, Corvallis, Oregon, USA

## Abstract

**Background:**

Large molecular sequence databases are fundamental resources for modern bioscientists. Whether for project-specific purposes or sharing data with colleagues, it is often advantageous to maintain smaller sequence databases. However, this is usually not an easy task for the average bench scientist.

**Results:**

We present the Personal Sequence Database (PSD), a suite of tools to create and maintain small- to medium-sized web-accessible sequence databases. All interactions with PSD tools occur via the internet with a web browser. Users may define sequence groups within their database that can be maintained privately or published to the web for public use. A sequence group can be downloaded, browsed, searched by keyword or searched for sequence similarities using BLAST. Publishing a sequence group extends these capabilities to colleagues and collaborators. In addition to being able to manage their own sequence databases, users can enroll sequences in BLASTAgent, a BLAST hit tracking system, to monitor NCBI databases for new entries displaying a specified level of nucleotide or amino acid similarity.

**Conclusion:**

The PSD offers a valuable set of resources unavailable elsewhere. In addition to managing sequence data and BLAST search results, it facilitates data sharing with colleagues, collaborators and public users. The PSD is hosted by the authors and is available at .

## Background

Bioscientists routinely interact with biological sequence databases. Often the interactions are with one of the several large national or international databases that provide web-based tools; for example, National Center for Biotechnology Information (NCBI) [1], EMBL Nucleotide Sequence Database [2] and DNA Data Bank of Japan [3]. To maintain data accuracy and integrity, well-defined procedures exist for submitting and changing entries in these databases. Although these procedures ensure a reliable resource for scientists around the world, they present significant restrictions to researchers who want to maintain their own sequences of interest in a web-accessible database that can be searched and shared with other users.

We present the Personal Sequence Database (PSD). The PSD is a suite of tools that allows a user to create, maintain and share small- to medium-size sequence databases. All interactions between a user and the database are over the internet with a web browser. Although at its core the PSD stores DNA and protein sequences, it also offers several data analysis tools, including ClustalW [4] and CAP3 [5], and the BLASTAgent, a custom tool that monitors NCBI sequence databases for new entries with sequence similarity to a particular PSD entry. Sequences in the PSD can be organized into groups that represent a user-defined level of association. The PSD allows a user to run BLAST [6] searches against the database sequences or against specific sequence groups within the database. Sequence groups can be maintained privately or shared with public users. If a sequence group is shared publicly, anyone can run BLAST searches against the sequences within the group. The PSD is maintained as a web-accessible public resource and is available, free of charge, to any educational user [7].

## Implementation

### User and database interfaces

The PSD user interface is generated by Perl (version 5.8.5) Common Gateway Interface (CGI) scripts. The scripts utilize the HTML::Template library (version 2.7) [8] to separate Perl code from the HTML markup. The web services are delivered by an Apache hypertext protocol daemon (httpd; Apache/1.3.29, mod_perl/1.29) [9,10] from a Sun Microsystems (Santa Clara, California, USA) V40z server running the Red Hat (Raleigh, North Carolina, USA) Linux operating system. The database tier is provided by the MySQL [11] database engine (version 3.23.58) from a separate, dedicated Sun Microsystems V40z running the Red Hat Linux operating system. Interactions between the scripts and the MySQL database are facilitated by a private ethernet network and the Perl DBI library (version 1.43) [12].

### NCBI sequence databases

Local versions of NCBI [1] DNA and protein databases are maintained by the Center for Genome Research and Biocomputing (CGRB). Once every 24 hours, custom Perl scripts download newly submitted sequences from NCBI and merge them into the local copies of each database. DNA and protein sequences submitted to NCBI within the last month are also maintained as separate databases. Periodically, new versions of the full databases are downloaded and replace the local merged versions. When necessary, individual sequences are extracted from these databases using tools distributed in the NCBI toolbox [13]; for example, fastacmd.

### BLAST searches

All BLAST searches use local NCBI BLAST [6] engines and local copies of NCBI sequence databases. BLAST searches are submitted to a local computer cluster and the results are parsed by custom Perl scripts.

### SOAP interface

The SOAP interface is facilitated by the SOAP::Lite Perl library (version 0.69) [14].

### Other software

The PSD makes use of various other software packages. Bioperl, version 1.4 [15], is used to manipulate biological sequences. Multiple sequence alignments are generated by ClustalW, version 1.8.4 [4]. CAP3 [5] is used to generate contigs from overlapping DNA fragments. Some metadata is compressed using the zlib library, version 1.1.4 [16].

## Results and Discussion

Although the PSD encompasses several distinct programs, users mainly interact with a handful of Perl CGI scripts (psd.cgi, psd_blast.cgi and bulkAddSeq.cgi) that access a MySQL database. Layers of abstraction render many of the underlying details invisible and users are presented with a consistent interface to perform database functions. We will describe the primary interface elements presented in a user's web browser.

### PSD registration

An online registration system is available from the main PSD website (see above). Upon successful registration the PSD is available for immediate use. During the registration, the user supplies a user name and password for login purposes. Subsequent changes to registration information are facilitated by a user administration interface.

### Sequence data entry

**Figure 1:**
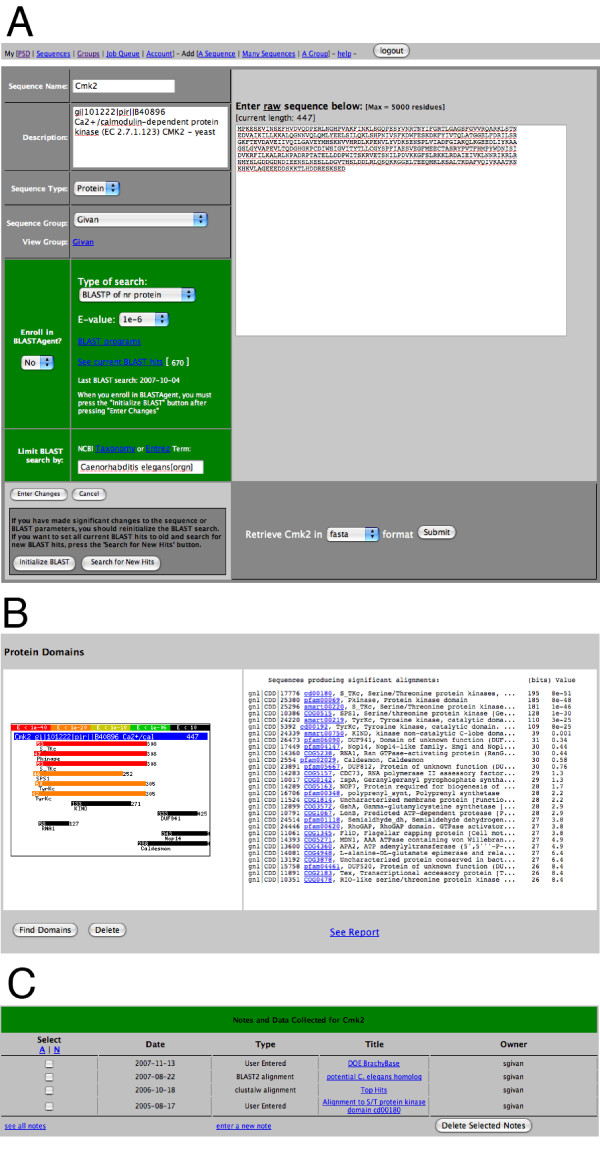
**PSD sequence data and metadata**. This is an example web page from the PSD containing all data and metadata associated with a protein sequence (DNA sequence pages are identical). A) Data entered by the user, either manually or automatically during batch import, and BLASTAgent settings. Notice that this particular sequence is not enrolled in BLASTAgent, but a BLASTP search of NCBI NR database has been run and the 596 hits are stored for the user to view. BLAST search options are illustrated; for example, the Entrez term "Caenorhabditis elegans [orgn]" limits the database hits to only those from *C. elegans*. B) Protein domains. Potential protein domains can be identified for any PSD sequence. A BLAST search of the NCBI CDD database is run and hits are illustrated graphically and by text. C) Sequence metadata. Sequence-associated data may be generated by tools integrated within the PSD, as in the top 2 entries, a BLAST2 alignment and a ClustalW multiple sequence alignment, or from external tools as the last entry illustrates.

The PSD allows sequence entry either individually or in batch. After logging in to the PSD, a user can enter a single sequence by following a link to "Add A Sequence." The raw sequence and associated metadata are entered in the appropriate areas of the page, see Figure 1A, which include: sequence name, description and sequence type, and the primary sequence. As described below, other options include enrolling the sequence in the BLASTAgent and identifying protein domains within the sequence. Upon entering as much information as desired, the entry is saved and the data becomes part of the user's database. An additional method is available to add a batch of multiple sequences. By following a link to "Add many sequences," the user is presented with a form to either upload a FASTA [17] file from their desktop computer or to copy and paste multiple sequences in FASTA format in a supplied box form element. Although not optimized for entering single sequences, the bulk form can also serve this purpose. In either case, sequence names and descriptions are extracted from the FASTA formatted sequence. Using either the single or bulk sequence entry forms, the sequences are available for immediate use once deposited into the PSD. Currently, public users are limited to 5000 sequences of up to 5000 residues each. These limitations may change in the future and can be negotiated by contacting the author. As described below, the limitations are designed to mitigate the computational demands of hosting the PSD but still provide adequate functionality for the average user.

### BLAST hit tracking system – BLASTAgent

An especially useful feature of the PSD is the ability to store results of BLAST searches. This process starts by initializing the BLASTAgent for a specific sequence. The user has the option of choosing from several combinations of BLAST [6] algorithm and NCBI [1] sequence database, including: BLASTN, TBLASTN, TBLASTX of non-redundant DNA; BLASTP or BLASTX of non-redundant protein; BLASTN or TBLASTN of GSS or STS; and BLASTN, TBLASTN or TBLASTX of mouse, human or other EST databases. All BLAST databases are maintained locally and updated nightly. After a BLAST search finishes, the results are viewable in a table format. This static list of BLAST hits remains available until the sequence is deleted from the user's database.

Subsequent to BLASTAgent initialization, it is possible to periodically re-run the BLAST search and identify new BLAST hits – ostensibly sequences added to the NCBI database since the last search. This process is either done manually, by pressing the "Search for New Hits" button, or automatically by choosing the "Yes" option for the form menu element entitled "Enroll in BLASTAgent?". If the BLASTAgent is activated, BLAST searches automatically run weekly. If new BLAST hits are identified the user will receive an email listing the sequences that have new hits. If no new BLAST hits are identified, the user will not receive an email message. Currently the user has no ability to alter when or how often the automatic BLASTAgent searches run, although this feature will be added in the future. However, a user has the option to re-run the BLASTAgent search manually at any time.

BLAST results can be filtered using two methods. First, a BLAST Expect value (E-value) limitation can be imposed. The E-value limit is always in effect, but the default value of 1^e^-06 can be modified by the user to allow either a higher or lower value. Current E-value choices range from 1^e^-100 to 100. Filtering by E-value is particularly effective at limiting the number of BLAST hits for sequences with high representation within the NCBI databases; for example, 16S ribosomal RNA genes. Users can also filter BLAST hits by NCBI Entrez terms. The PSD can use any valid Entrez term to filter BLAST hits. Filtering by Entrez query is especially effective at targeting a specific set of sequences; for example, sequences from a particular organism. The Entrez vocabulary is available from the NCBI website [18] or from links within the PSD.

### BLAST hit sequence manipulation

If a BLAST search identifies hits for a PSD sequence, several options are available to manipulate the hit sequences. When viewing the BLAST results, links are provided to the specific entry at NCBI and to display the BLAST alignment of the query sequence and the database hit sequence. Additional analysis options are available depending on the type of BLAST database searched. For example, if the hits are from a protein database, specific hits can be selected and subjected to a ClustalW multiple sequence alignment. If the hits are from a DNA database they can be subjected to either a ClustalW or CAP3 multiple sequence alignment algorithm. All database hits are available for direct download in FASTA format irrespective of sequence type. BLAST, ClustalW and CAP3 alignments can be saved as metadata associated to the PSD sequence used as the query in the BLAST search (see below).

### PSD sequence domain identification

Any PSD sequence can be subjected to protein domain identification. Domain identification is facilitated by a BLAST search of local copy of the NCBI Conserved Domain Database (CDD) [19]. A BLASTP or BLASTX search is used depending on whether the PSD sequence is a protein or DNA sequence, respectively. Results of the CDD BLAST search are saved both as text and as a graphical representation of the domain hits relative to the query sequence, see Figure 1B. Links to the NCBI web page for each domain are provided in the text output. The results of a domain search remain associated with the PSD sequence entry until the domain search is deleted or the PSD sequence entry is removed from the database.

### PSD sequence notes

Metadata, referred to as "Sequence Notes", can be associated and saved with any PSD sequence entry, see Figure 1C. These metadata can be hand-entered text, a BLAST alignment, or a multiple sequence alignment generated by ClustalW or CAP3 (see above). When a sequence alignment is generated within the PSD, it is presented as a web page that can be permanently associated with the PSD sequence entry. If saved, the graphical nature of the alignments as well as the text output are faithfully stored until they are manually deleted or the PSD sequence entry is removed from the database. Additionally, an interface is provided to manage the notes; for example, changing a note title or deleting batches of notes.

### PSD sequence groups

Often it is useful to collect sequences related to each other by some characteristic. The PSD facilitates this process through the creation of sequence groups. Any PSD user can create an unlimited number of sequence groups. Individual sequences are submitted to a group using checkboxes and a pull-down menu to select the desired group. If the sequences are added in bulk (see above), the user can select a group to which the sequences will be added upon submission of the form. An interface is available to add or delete sequences from a group or to change the group membership of sequences from one group to another. Additionally, group characteristics can be changed using the Sequence Group Administration (SGA) page. Sequence groups are a powerful way to organize sequences for further analysis; for example, BLAST searches or sequence sharing (see below).

### BLAST searches of PSD databases

Any user can search their PSD using BLAST. If a user has created sequence groups, these can be searched independently from the other sequences in the database. All of the BLAST algorithms are available from a pull-down menu on the BLAST interface. Before the BLAST search occurs, the PSD collects the sequences appropriate for the BLAST algorithm; for example, DNA sequences for a BLASTN search or protein sequences for a BLASTP search. The collected sequences are converted to a BLAST database, the BLAST search runs on a PSD server and the data are delivered to the user's browser. Multiple query sequences can be submitted at once – the BLAST output is concatenated. Each BLAST search is accompanied by a graphical representation of the BLAST hits relative to the query sequence. Users can easily move between BLAST results and PSD sequence entries using links back to the PSD in the BLAST results.

### Sharing PSD sequence groups

Using the SGA page (see above), any PSD sequence group can be made publicly available, which is described as "published" within the PSD. All data associated with published PSD sequences are available for browsing by public users, including: the sequence, sequence notes, protein domain information and BLAST hits, if available. However, public users cannot alter any characteristic of the sequence or associated metadata. The published sequence groups are available through a common interface specialized for this purpose. A link to the public interface, which can be distributed to collaborators or displayed on a web page, is provided on the SGA page when the group is published. The SGA page allows a user to provide optional text that will be rendered in HTML on the public interface. This optional text can be used to provide links to other resources the user feels is appropriate or valuable for the specific sequences contained within the group. Any public sequence group can easily be made private again using the SGA page.

In addition to the shared sequences and associated metadata, public PSD sequence groups are available for BLAST searches by public users. A link to the public BLAST search page is provided on the public interface. When public users follow this link, they are presented with a simple BLAST form that can be submitted with a query sequence in FASTA format. The BLAST results contain links back to the sequences in the published sequence group of the PSD. Using the SGA page, a user can supply a template for links to an external resource; for example, an external database containing more extensive information about sequences within the group. These external links will be displayed for every hit in the BLAST report. External links require a specialized syntax, which is explained on the SGA page.

Each public sequence group has its own home web page. Thus, users can maintain multiple independent public sequence groups, each available as a distinct shareable and/or public resource. As mentioned above, there is no limit to the number of sequence groups that can be created by a single user.

### Simple Object Access Protocol (SOAP) interface

A SOAP interface to public sequences (see above) is available for users interested in writing custom software to interact remotely with the PSD. Full PSD functionality has not been included in the SOAP interface, but this is a future goal. For detailed information about using the SOAP interface, users are directed to the PSD information website [7].

### Computational requirements and considerations

From the perspective of providing a public resource such as the PSD, an obvious concern is the potential computational requirements needed. Although still a concern, several mechanisms have been devised to minimize the computational load. There are two main components to the computational load: storage space and server Central Processing Unit (CPU) usage. Multiple strategies reduce the storage space requirements for the MySQL tables storing the PSD data. The most obvious strategy, as described above, is to limit the number and length of sequences a user can store in a particular PSD. These limitations are first approximations and may be altered in the future as space requirements are re-evaluated. Another strategy is to not store BLAST alignments in the database. The necessary information to recreate a particular BLAST alignment on the fly using bl2seq (NCBI) is stored in the PSD, alleviating storage of the actual alignment text. Finally, when a user stores metadata, which is often a web page, the data are subjected to a zlib compression algorithm [16] to minimize their size within the database.

Currently, the PSD contains 101,496 sequence entries with a length of less than 5000 residues. Given the above sequence constraints, this represents approximately 20 users if each user stored the maximum of 5000 sequences. To store these sequences, the database engine requires approximately 84MB of disk space. About 1% of these sequences are enrolled in BLAST searches, which increases storage requirements by about 100 MB. Given these parameters, we estimate that PSD usage could increase 4000-fold and still be rather inexpensive to manage at current hard drive prices. It is expected that the cost per byte of disk space will continue to drop, increasing the manageable number of potential PSD users.

As mentioned above, another concern is CPU usage. Three aspects of the PSD affect processor load: the Apache web delivery engine [10], the MySQL database engine, and the BLAST searches. The most fundamental strategy to manage these loads is to distribute the services to distinct machines specialized for their purpose. Demands on the PSD web server are manageable and are less of a concern than the MySQL and BLAST CPU requirements. Currently the MySQL database is hosted by a single four-processor machine with 4GB of RAM. It is likely that this will be the weak link within the PSD architecture. Imposing the above limits on an individual user's PSD will help to manage MySQL load, but depending on its popularity the PSD could easily outgrow the current MySQL machine. If necessary, multiple MySQL servers could be incorporated into the architecture to provide parallel and redundant database servers. As database load gradually increases over time, additional database servers can be added. Several strategies are used to manage the computational demands imposed by the BLAST searches. The PSD runs three types of BLAST searches. When a user initializes a BLASTAgent an initial BLAST search runs against a full NCBI database; i.e., NT or NR. These BLAST searches are quite demanding and usually last for several minutes. Alternatively, when identifying new hits, BLAST searches most often run against databases that contain only those sequences deposited into NCBI within the last month. Therefore, these are smaller databases and the BLAST searches are much less demanding, usually lasting only several seconds. The majority of BLAST searches are of the latter type, which minimizes the computational demands. BLAST searches can also be run against a user's database. Due to the size limitation imposed on individual PSD databases, these BLAST searches are quite fast and do not require significant CPU time. In addition to the above measures, a custom-designed daemon process running on a PSD server manages jobs to limit the number running at any given time, which mitigates the computational demand. Finally, no method exists to bulk-submit sequences to the BLASTAgent system. Although seemingly a drawback, this design is intentional and requires a user to decide which sequences are most important for BLAST searches and minimizes inefficiencies associated with running BLAST searches with many highly similar PSD entries.

The PSD is currently supported by the CGRB at Oregon State University. The CGRB maintains an extensive computational infrastructure, including a computer cluster that the PSD leverages to run BLAST searches and multiple sequence alignments. Several nodes within the cluster are dedicated to the PSD and additional nodes can be added when necessary. We estimate that the number of BLAST searches could increase 100-fold and still remain manageable. The PSD represents an important public resource and the CGRB is committed to supporting it into the foreseeable future.

## Conclusion

The Personal Sequence Database is an integrated suite of tools that allows users to create and maintain small- to medium-sized biological sequence databases. Multiple requisite database functions exist, including: sequence uploading and downloading, database searching by keyword and BLAST sequence similarity searching. The PSD provides several sequence analysis functions, including protein domain identification and multiple sequence alignments. The PSD also offers BLASTAgent, a powerful resource that provides an automated system for tracking new BLAST hits appearing in NCBI databases. Although some shared functionality exists with other software [20-26], the PSD represents a unique set of resources that is unavailable from any other single location.

A user can organize database entries into sequence groups, ostensibly representing some shared relationship. Sequence groups behave as individual units within the larger database. Each sequence group can be maintained privately or publicly. Public sequence groups, also referred to as "published" groups within the PSD, are available to any user as a read-only resource. As such, a published group represents an individual web-accessible sequence database that can be distributed to colleagues (ie, as a URL) or maintained as a long-term public entity. Therefore, any single user can maintain multiple sequence groups, each of which can be either private or published. An interesting use of these capabilities is to incorporate PSD sequence groups into other web resources. For example, the Marine Microbial Genomics (MMG) website [27] uses PSD sequence groups to allow visitors to run BLAST searches against the genomes of dozens of newly sequenced bacterial genome sequences. Although the MMG website is maintained in association with the authors, anyone could mimic this method on their own sites with no involvement of the author. Therefore, the PSD can provide computationally-intensive resources to researchers within less well-developed computational infrastructures.

It is common for a geographically distributed group of researchers to collaborate on large projects. Often these projects revolve around accumulating DNA sequences, either for genome assembly, gene discovery or expression analysis. For these types of projects, the PSD can facilitate research and data sharing between groups by serving as a central resource to which all groups can refer for sequence data. Although in its current version the PSD does not allow multiple users to have read/write access to individual databases, future versions will include this capability. Interested parties can contact the authors for more information about this functionality.

Finally, there are several areas of additional functionality scheduled for future PSD releases. These include more extensive search capabilities, an advanced BLAST interface, more configuration options (ie, when and how often BLASTAgent runs), downloadable BLAST results, automatic determination of Gene Ontology [28] terms associated with user-specified entries and a more extensive collection of sequence analysis tools, including PFAM searches [29] and basic phylogeny tools. These improvements will appear gradually as development proceeds. Interested parties should refer to the PSD information web page for future announcements.

## Availability and requirements

**Project name: **The Personal Sequence Database

**Project home page: **

**Operating system: **Platform independent

**Programming language: **Perl, SQL, Javascript

**Other requirements: **Online registration – 

**License: **GNU General Public License

**Any restrictions to use by non-academics: **Contact corresponding Author

## Authors' contributions

SAG conceived project and contributed all Perl and Javascript code and designed complete database schema, and provided test-case scenarios. CMM designed, tested and optimized all computational and network hardware and provided test-case scenarios. JCC contributed to infrastructure development and provided test-case scenarios. All authors reviewed and approved manuscript.
